# Supplementary material for: Naturally-occurring spinosyn A and its derivatives function as argininosuccinate synthase activator and tumor inhibitor
Source: Nat Commun. 2021 Apr 15;12:2263. doi: 10.1038/s41467-021-22235-8 (PMC8050083; doi:10.1038/s41467-021-22235-8)
Supplement: Supplementary file 1 — Supplementary Information [file 41467_2021_22235_MOESM1_ESM.pdf]

Supplementary Information

Naturally-ocurring spinosyn A and its derivatives function as argininosuccinate synthase activator and tumor inhibitor

Table of contents

Ⅰ. Supplementary tables

1. Supplementary Table 1: Clinicopathological data breast cancer samples.

2. Supplementary Table 2: Primers list.

Ⅱ. Supplementary figures

1. Supplementary Figure 1: Synthesis schemes for LM-2I (**2**) and its intermediates.

2. Supplementary Figure 2: Synthesis scheme for SPAH (**3**).

3. Supplementary Figure 3: Synthesis schemes for probe and its intermediates.

4. Supplementary Figure 4: Dose response curves of breast cancer cell lines to SPA and LM-2I treatment.

5. Supplementary Figure 5: Effect of SPA and LM-2I on tumor growth in mouse xenograft model.

6. Supplementary Figure 6: Discovery and confirmation of SPA and LM-2I target(s).

7. Supplementary Figure 7: SPA and LM-2I exhibit anti-tumor activity by targeting ASS1.

8. Supplementary Figure 8: SPA and LM-2I inhibit pyrimidine synthesis.

9. Supplementary Figure 9: ^{1}H , ^{13}C NMR and MS spectra for LM-2I (**2**).

10. Supplementary Figure 10: ^{1}H , ^{13}C NMR and MS spectra for SPAH (**3**).

11. Supplementary Figure 11: ^{1}H , ^{13}C NMR and MS spectra for Biotin-SPA (**4**).

12. Supplementary Figure 12: ^{1}H , ^{13}C NMR and MS spectra for Biotin-SPAH (**5**).

13. Supplementary Figure 13: ^{1}H , ^{13}C NMR and MS spectra for **7**.

14. Supplementary Figure 14: ^{1}H , ^{13}C NMR and MS spectra for **8**.

15. Supplementary Figure 15: ^{1}H , ^{13}C NMR and MS spectra for **9**.

Ⅰ. Supplementary tables

Supplementary Table 1. Clinicopathological data of breast cancer samples

	No. of cases	No. of recurrence (%)	No. of no recurrence (%)
Age (years)			
< 40	16	6 (10.0)	10 (12.2)
40-49	65	29 (48.3)	36 (43.9)
50-59	41	18 (30.0)	23 (28.0)
60-69	18	6 (10.0)	12 (14.6)
≥70	2	1 (1.7)	1 (1.2)
Total	142	60 (100.0)	82 (100.0)
ER			
Negative	40	20 (33.9)	20 (24.1)
Positive	102	39 (66.1)	63 (75.9)
Total	142	59 (100.0)	83 (100.0)
PR			
Negative	48	24 (40.7)	24 (28.9)
Positive	94	35 (59.3)	59 (71.1)
Total	142	59 (100.0)	83 (100.0)
HER2			
Negative	86	39 (66.1)	47 (59.5)
Positive	52	20 (33.9)	32 (40.5)
Total	138	59 (100.0)	79 (100.0)

Supplementary Table 2. Primers list.

Gene name	Assay	Sense	Sequence
Human Wild type (WT) *ASS1*	Prokaryotic expression	Forward	ATGGGTCGCGGATCCGAATTCATGTCCAGCAAAGGCTCC
		Reverse	CTCGAGTGCGGCCGCAAGCTTTTATTTGGCAGTGACCTT
uman *ASS1* Cys19Ala	Prokaryotic expression	Forward	GACACCTCGGCCATCCTCGTGTGGCTGAAGGAACAAGGCT
		Reverse	ACGAGGATGGCCGAGGTGTCCAGGCCGCCACTGTAGGCCA
Human *ASS1* Cys97Asp	Prokaryotic expression	Forward	GCCAGGCCCGACATCGCCCGCAAACAAGTGGAAATCGCCC
		Reverse	CGGGCGATGTCGGGCCTGGCAAGAGAGGTGCCCAGGAGGT
Human *ASS1* Cys132Ala	Prokaryotic expression	Forward	GAGCTCAGCGCCTACTCACTGGCCCCCCAGATAAAGGTCA
		Reverse	AGTGAGTAGGCGCTGAGCTCAAACCGGACCTGATCGTTCC
Human *ASS1* Cys331Ala	Prokaryotic expression	Forward	AGCCCTGAGGCTGGATTTGTCCGCCACTGCATCGCCAAGT
		Reverse	ACAAATCCAGCCTCAGGGCTGTGCCAGAAACCGGTATACA
Human *ASS1* Cys337Ala	Prokaryotic expression	Forward	GTCCGCCACGCCATCGCCAAGTCCCAGGAGCGAGTGGAAG
		Reverse	TTGGCGATGGCGTGGCGGACAAATCCACACTCAGGGCTGT
Human *ASS1*	Prokaryotic	Forward	GTCCGCCACGCCATCGCCAAGTCCCAGGAGCGAGTGGAAG

Cys337Ala	expression	Reverse	TTGGCGATGGCGTGGCGGACAAATCCACACTCAGGGCTGT
ASS1- sgRNA1	CRISPR/Cas9	Forward	CACCGCAGCCACACGAGGATGCACG
		Reverse	AAACCGTGCATCCTCGTGTGGCTGC
ASS1- sgRNA2	CRISPR/Cas9	Forward	CACCGCGAGGATGCACGAGGTGTCC
		Reverse	AAACGGACACCTCGTGCATCCTCGC

Ⅱ. Supplementary figures

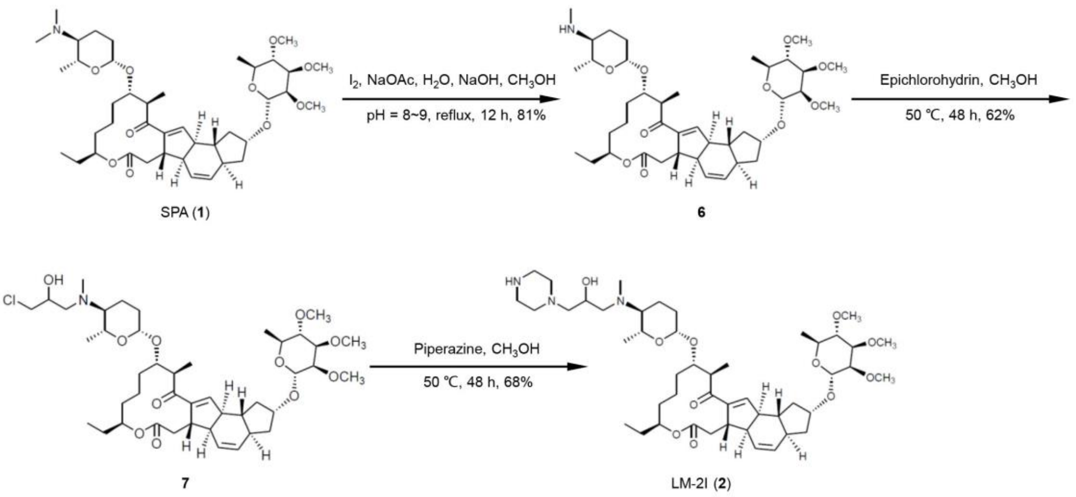

Supplementary Figure 1 | Synthesis schemes for LM-2I (2) and its intermediates.

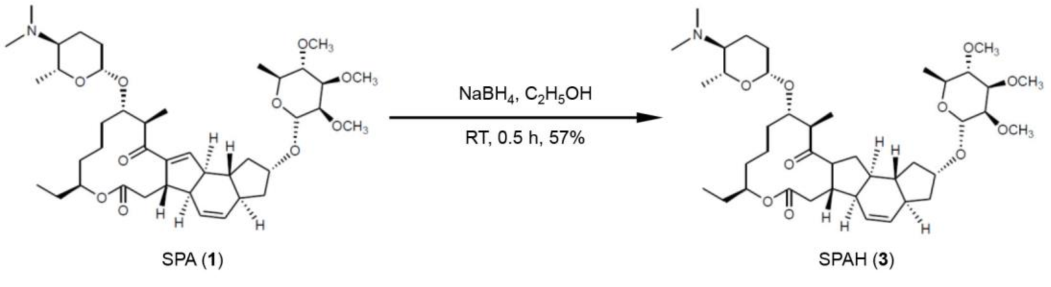

Supplementary Figure 2 | Synthesis scheme for SPAH (3).

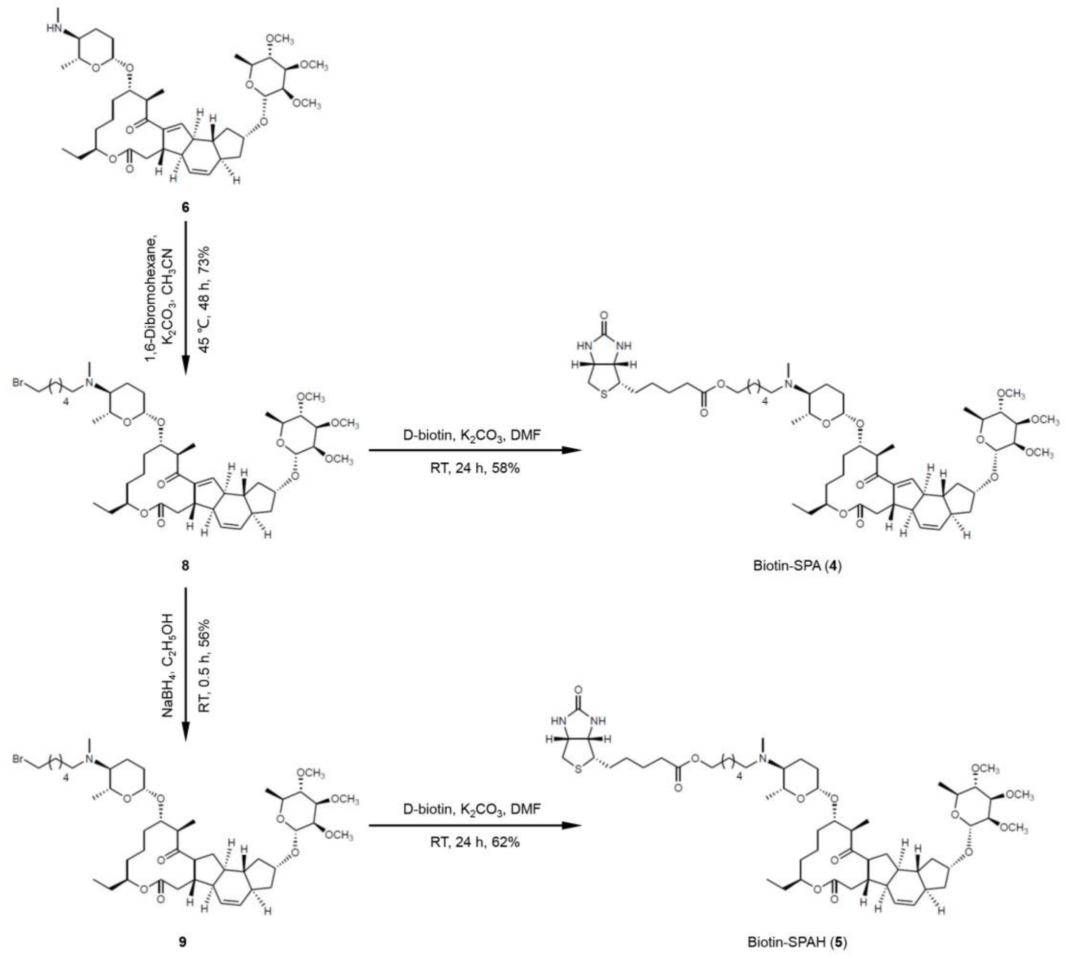

Supplementary Figure 3 | Synthesis schemes for probe and its intermediates.

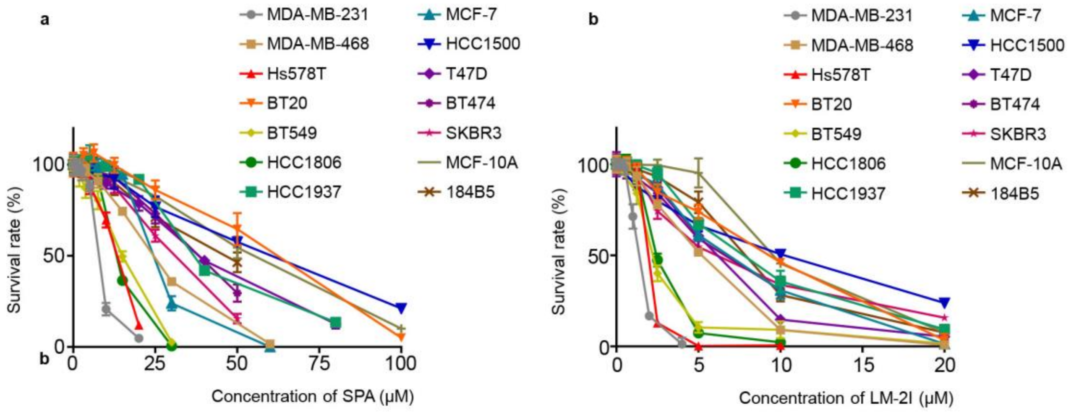

Supplementary Figure 4 | Dose response curves of breast cancer cell lines to SPA and LM-2I treatment. Crystal violet assay for 14 breast cancer cell lines treated with different concentrations of SPA (a) and LM-2I (b) for 48 h (mean ± s.d. , n = 3

biologically independent experiments) **.** Source data are provided as a Source Data file.

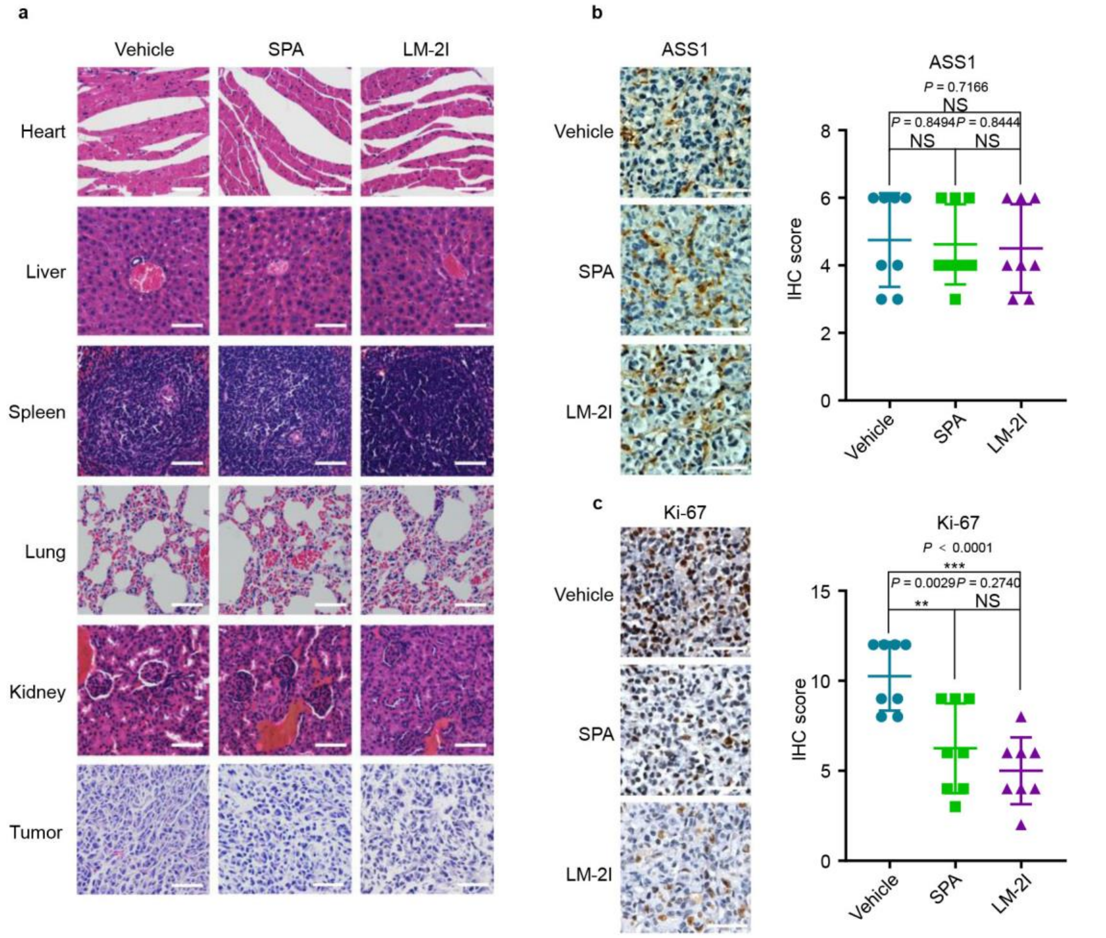

Supplementary Figure 5 | Effect of SPA and LM-2I on tumor growth in mouse xenograft model. MDA-MB-231 cells were inoculated subcutaneously into the right flank of nu/nu female mice. When the average tumor size reached about 100 mm^3, mice were treated with ether vehicle, SPA (10 mg/kg/day) or LM-2I (5 mg/kg/day). 28 days later, mice were sacrificed and tumors as well as mouse organs were collected. The paraffin-embedded heart, liver, spleen, lung, kidney, and tumor tissue sections were stained with hematoxylin and eosin (a) (*n* = 8 mice per group) **.** Scale bar represents 50 µm. (b, c) Representative imagines of IHC and quantitative data for the expression of

ASS1 (b) and Ki-67 (c) protein in tumor tissues. Scale bar represents 50 μm. (Data are presented as mean ± s.d., n=8) . ** $P < 0.01$, *** $P < 0.001$, NS: not significant ($P > 0.05$) from two-tailed unpaired Student's *t*-tests. Source data are provided as a Source Data file.

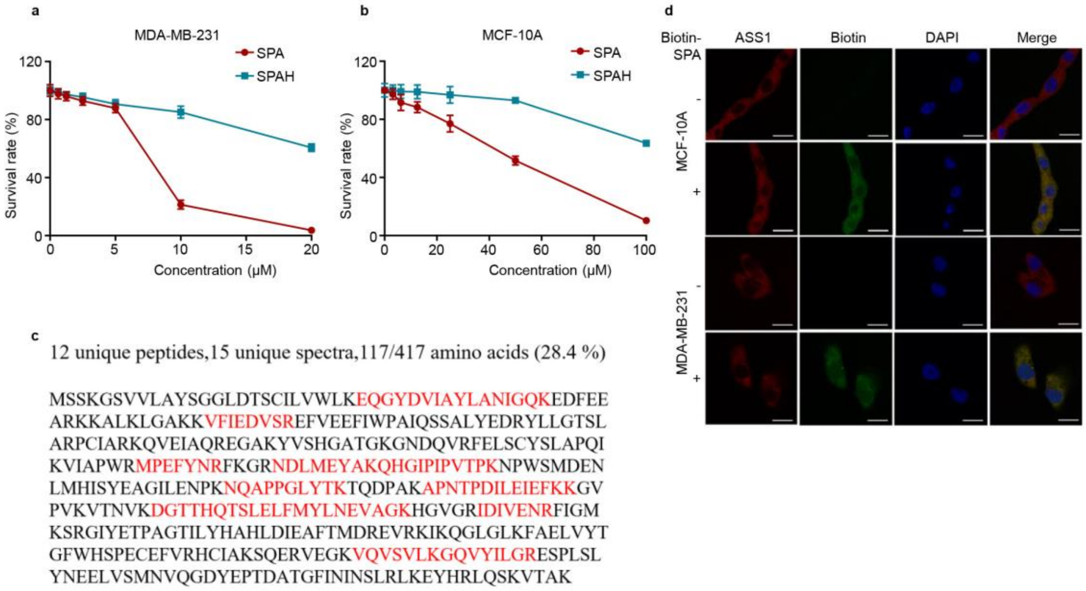

Supplementary Figure 6 | Discovery and confirmation of SPA and LM-2I target(s). (a,b**)** Crystal violet assay for the viability of MDA-MB-231 (a) and MCF-10A (b) cells treated with SPAH and SPA for 48 h (mean ± s.d. , $n = 3$ biologically independent experiments) . (c) The protein band in experiments of Fig. 2e was excised, de-stained, and subjected to in-gel tryptic digestion and analysis by MS. The identified ASS1 peptides are in red. (d) Co-localization of Biotin-SPA with ASS1. MDA-MB-231 and MCF-10A cells were treated with or without Biotin-SPA for 4 h, and then stained with ASS1 antibody (red) and streptavidin-FITC (green) , followed by counterstaining with DAPI ($n = 3$ biologically independent experiments) . Scale bars are 20 μm. Source data are provided as a Source Data file.

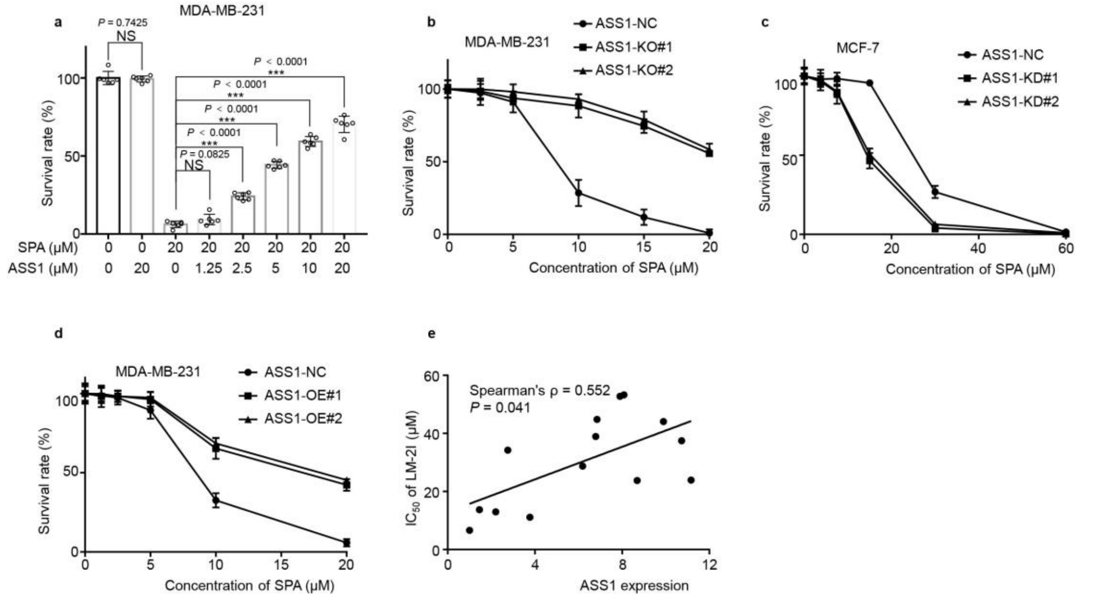

Supplementary Figure 7 | SPA and LM-2I exhibit anti-tumor activity by targeting ASS1. (a) Crystal violet assay for the viability of MDA-MB-231 cells treated with SPA or SPA pre-incubated with different concentrations of ASS1 recombinant protein for 48 h (mean ± s.d. , n = 6 biologically independent experiments) , *** P < 0.001 from two-tailed unpaired Student's t-tests. (b) Crystal violet assay for the viability of control and ASS1-KO MDA-MB-231 cells treated with different concentrations of SPA (mean ± s.d. , n = 3 biologically independent experiments). (c) Crystal violet assay for the viability of control and ASS1-KD MCF-7 cells treated with SPA (mean ± s.d. , n = 3 biologically independent experiments) . (d) Crystal violet assay for the viability of control and ASS1-OE MDA-MB-231 cells treated with SPA (mean ± s.d. , n = 3 biologically independent experiments) . (e) The correlation between the relative ASS1 expression in cells and the IC_{50} of these cells to SPA (n = 3 independent experiments from n = 14 cell lines). P values of mean ± s.d. were determined by Pearson Coefficient. Source data are

provided as a Source Data file.

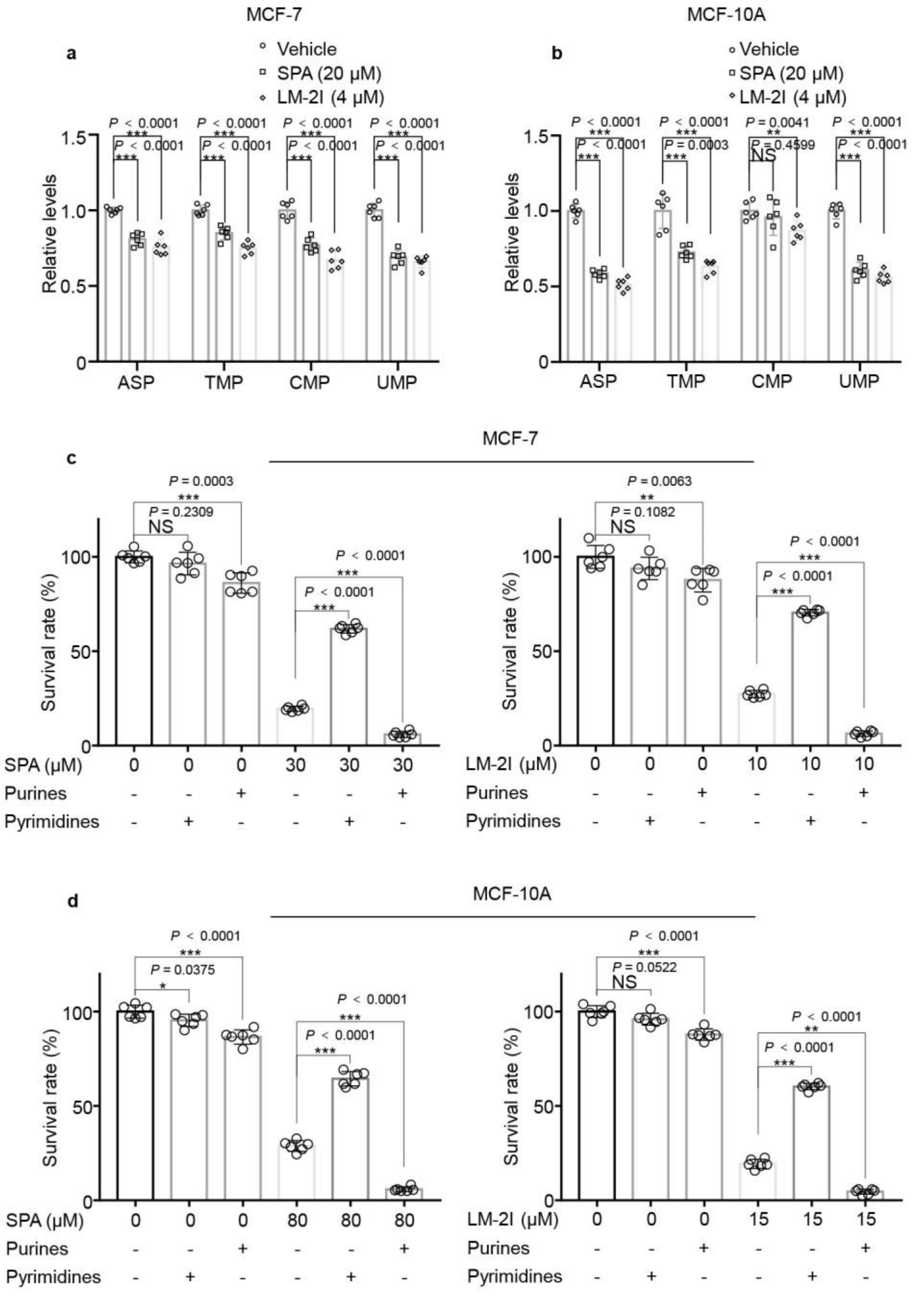

Supplementary Figure 8 | SPA and LM-2I inhibit pyrimidine synthesis. (a, b) LC-MS analysis of ASP, UMP, CMP and TMP levels in MCF-7 (a) and MCF-10A (b) cells treated with SPA and LM-2I for 24 h (mean ± s.d. , n = 6 biologically independent experiments) . ** P < 0.01, *** P < 0.001, NS: not significant (P > 0.05) from two-tailed

unpaired Student's *t*-tests. (c,d) Crystal violet assay for MCF-7 (c) and MCF-10A (d) cells treated with SPA (left panel) or LM-2I (right panel) in the absence or presence of pyrimidines or purines for 48 h (mean ± s.d. , $n = 6$ biologically independent experiments) . * $P < 0.05$, ** $P < 0.01$, *** $P < 0.001$, NS: not significant ($P > 0.05$) from two-tailed unpaired Student's *t*-tests. Source data are provided as a Source Data file.

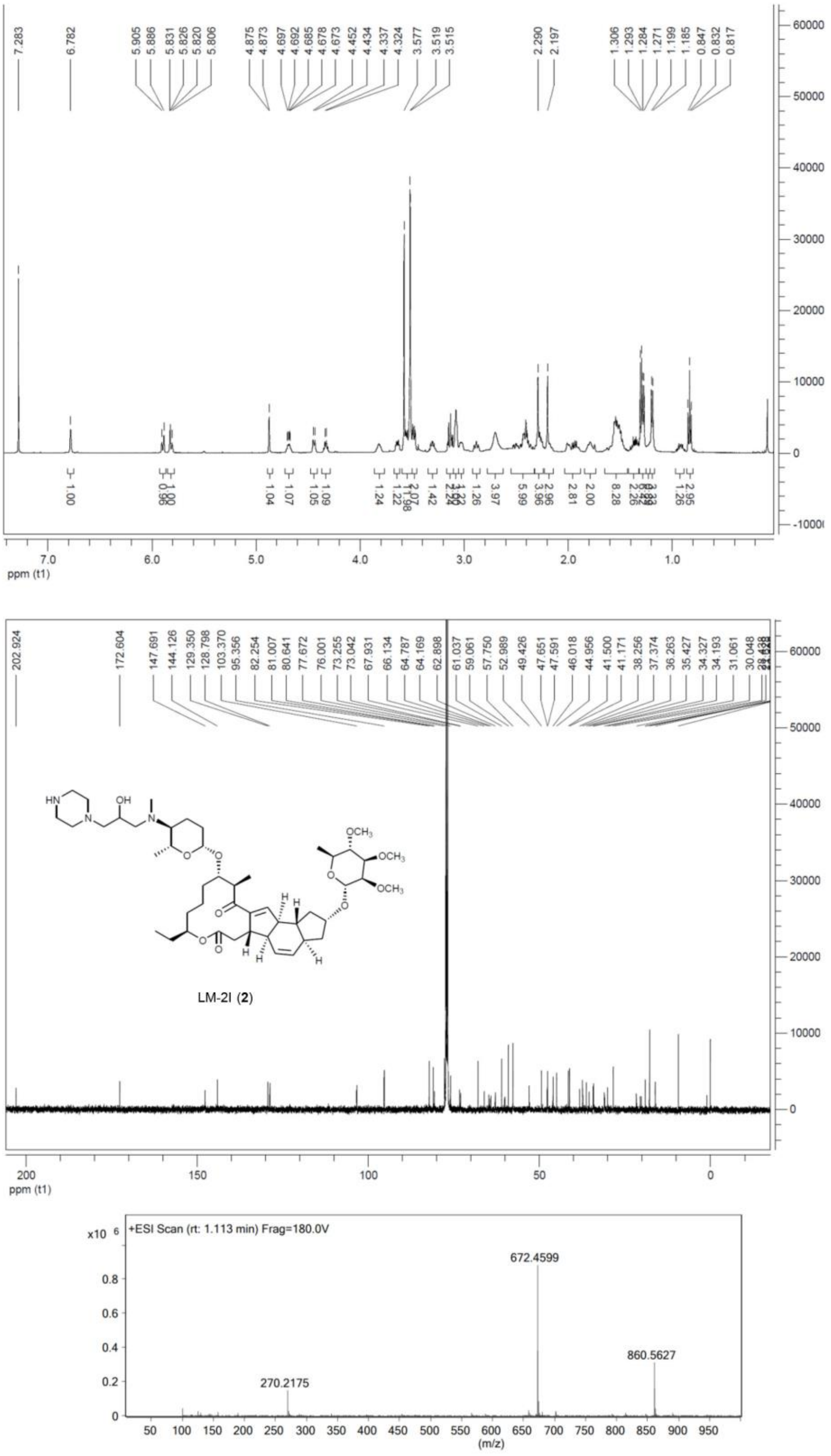

Supplementary Figure 9 | ^{1}H , ^{13}C NMR and MS spectrum for LM-2I (2).

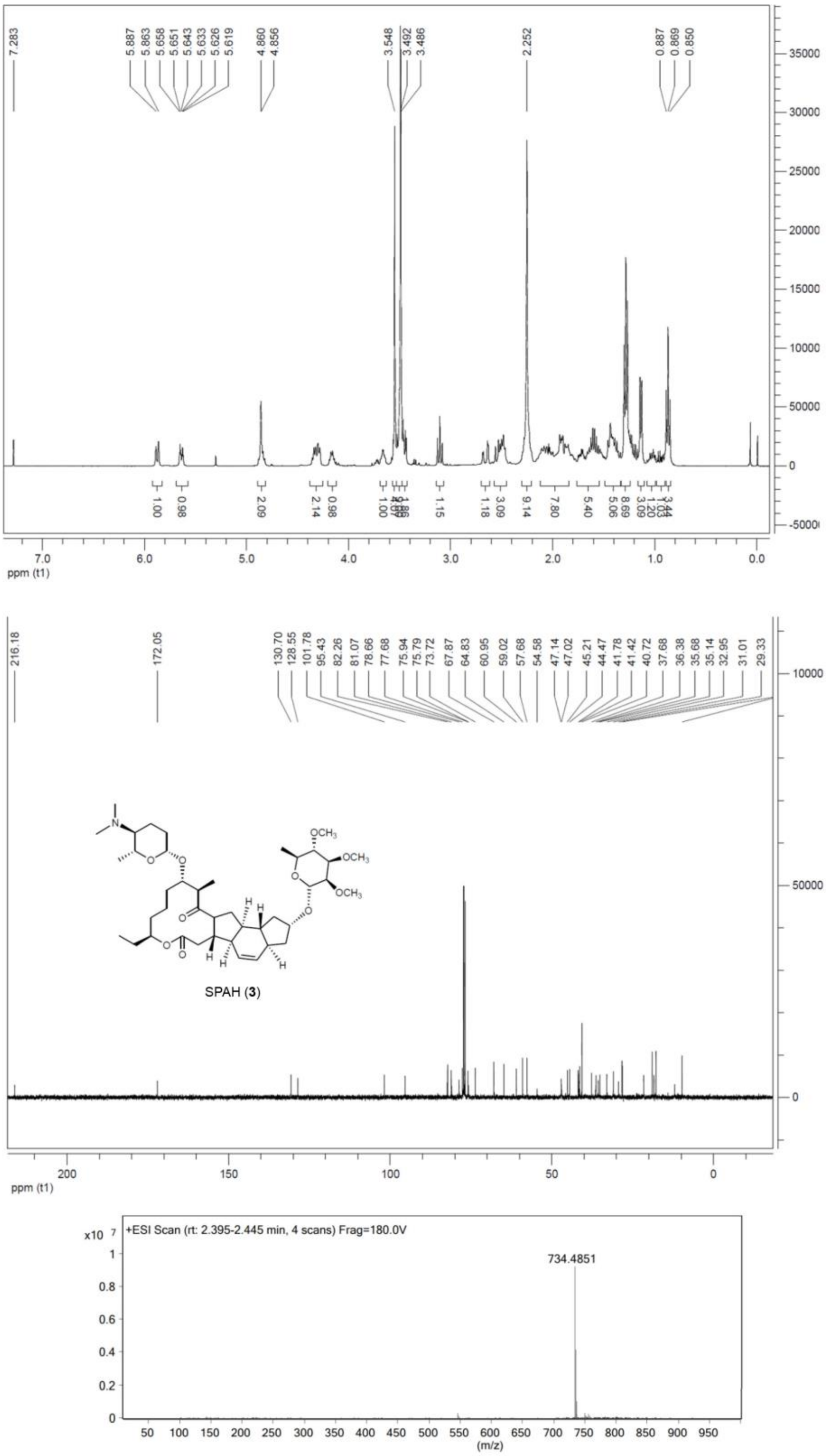

Supplementary Figure 10 | ^{1}H , ^{13}C NMR and MS spectrum for SPAH (3).

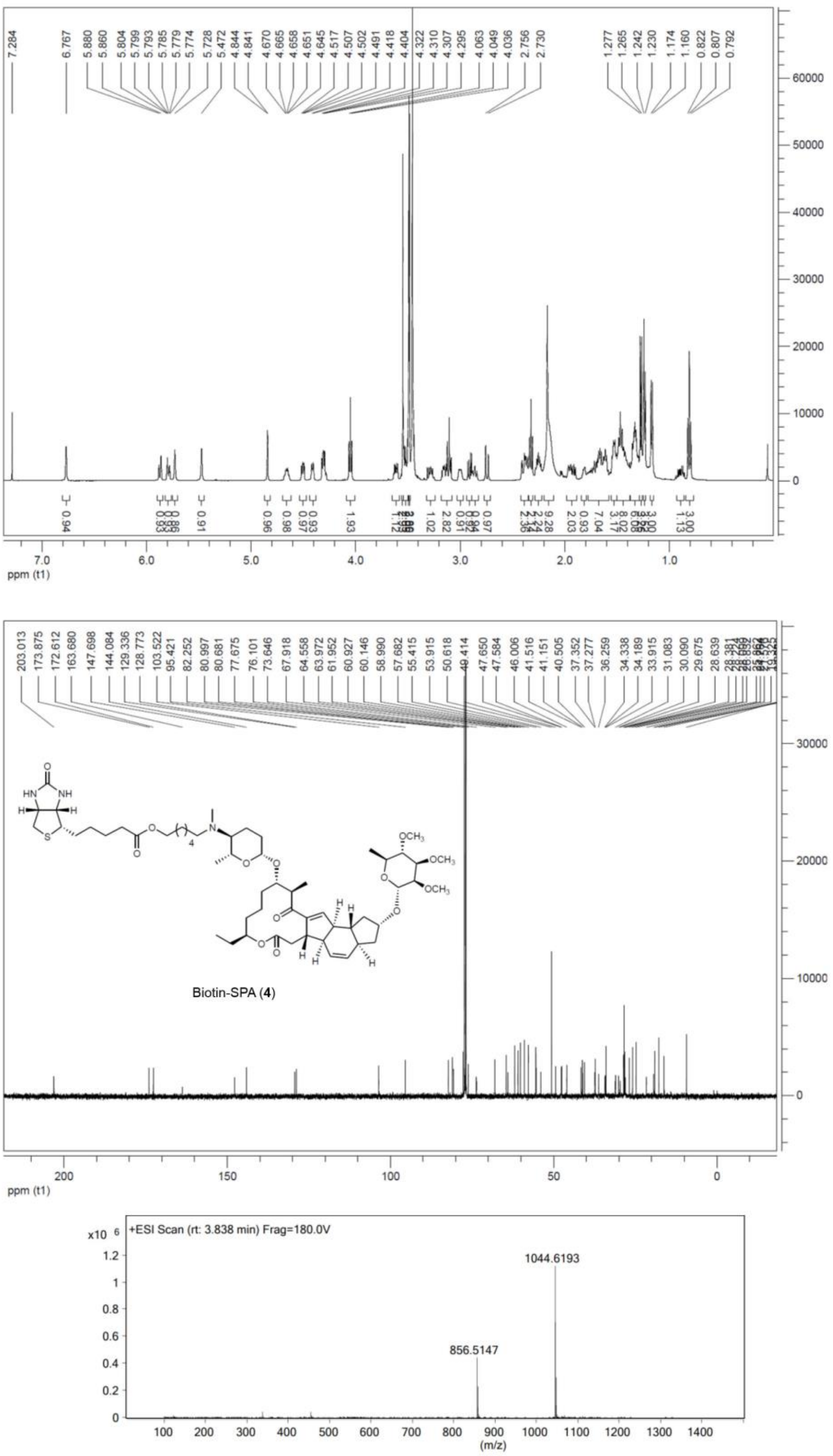

Supplementary Figure 11 | ^{1}H , ^{13}C NMR and MS spectrum for Biotin-SPA (4).

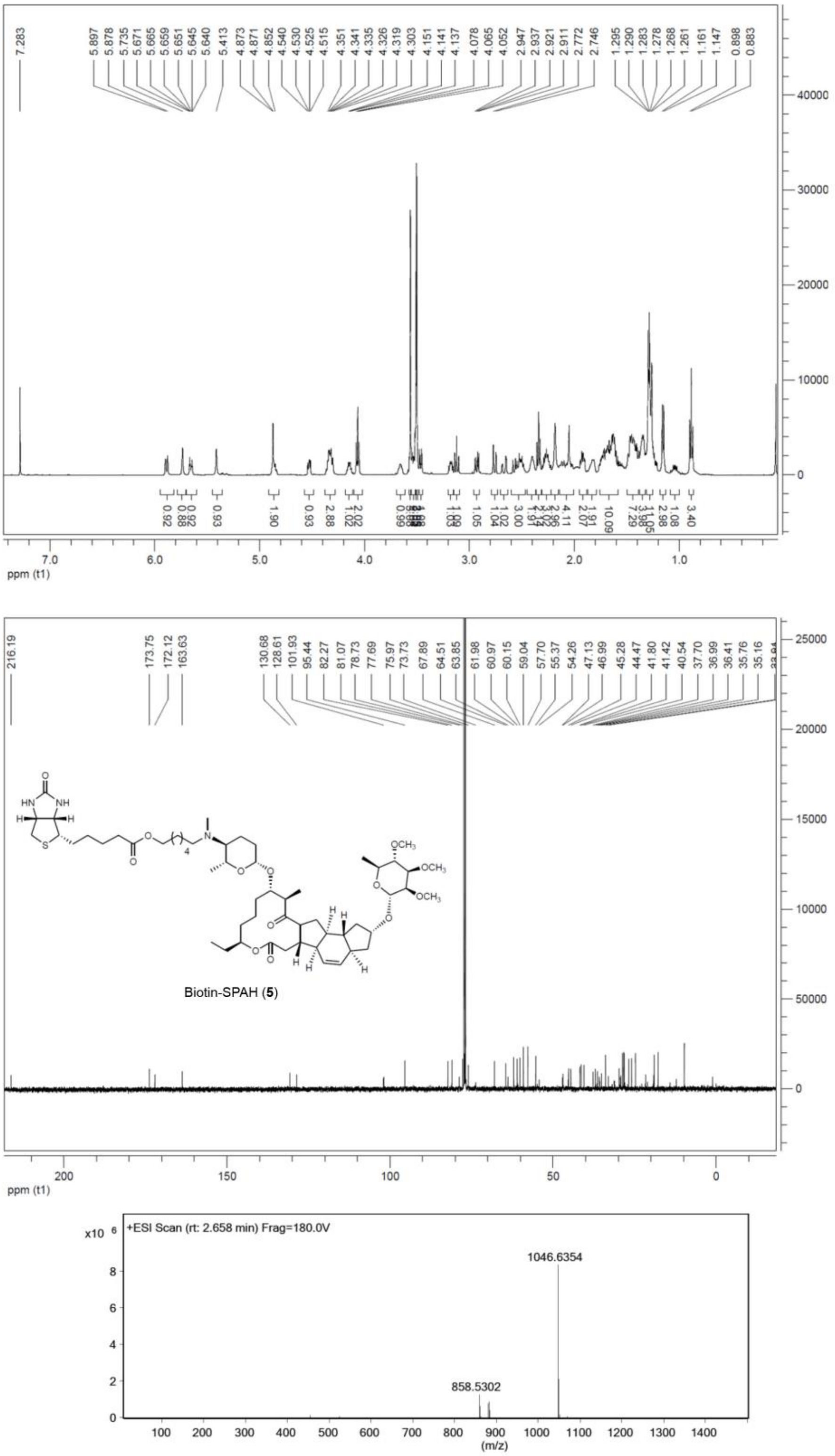

Supplementary Figure 12 | ^{1}H , ^{13}C NMR and MS spectrum for Biotin-SPAH (5).

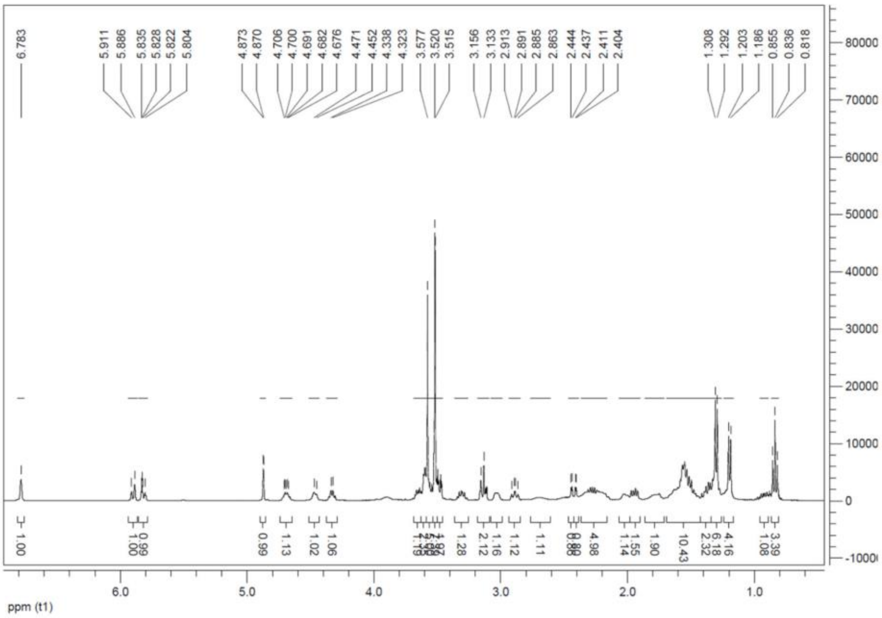

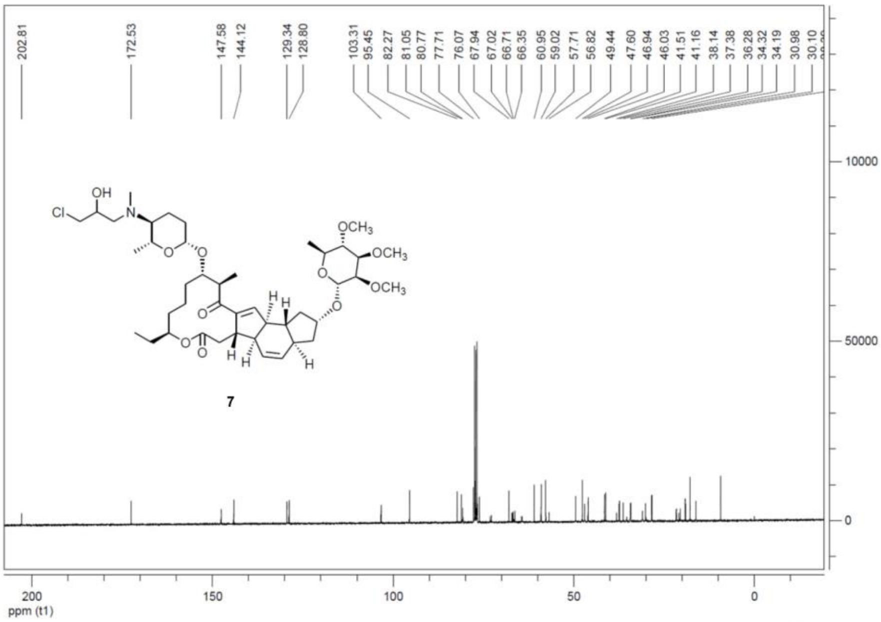

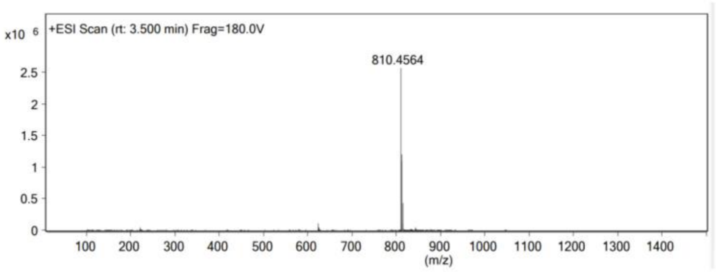

Supplementary Figure 13 | ^{1}H , ^{13}C NMR and MS spectrum for 7.

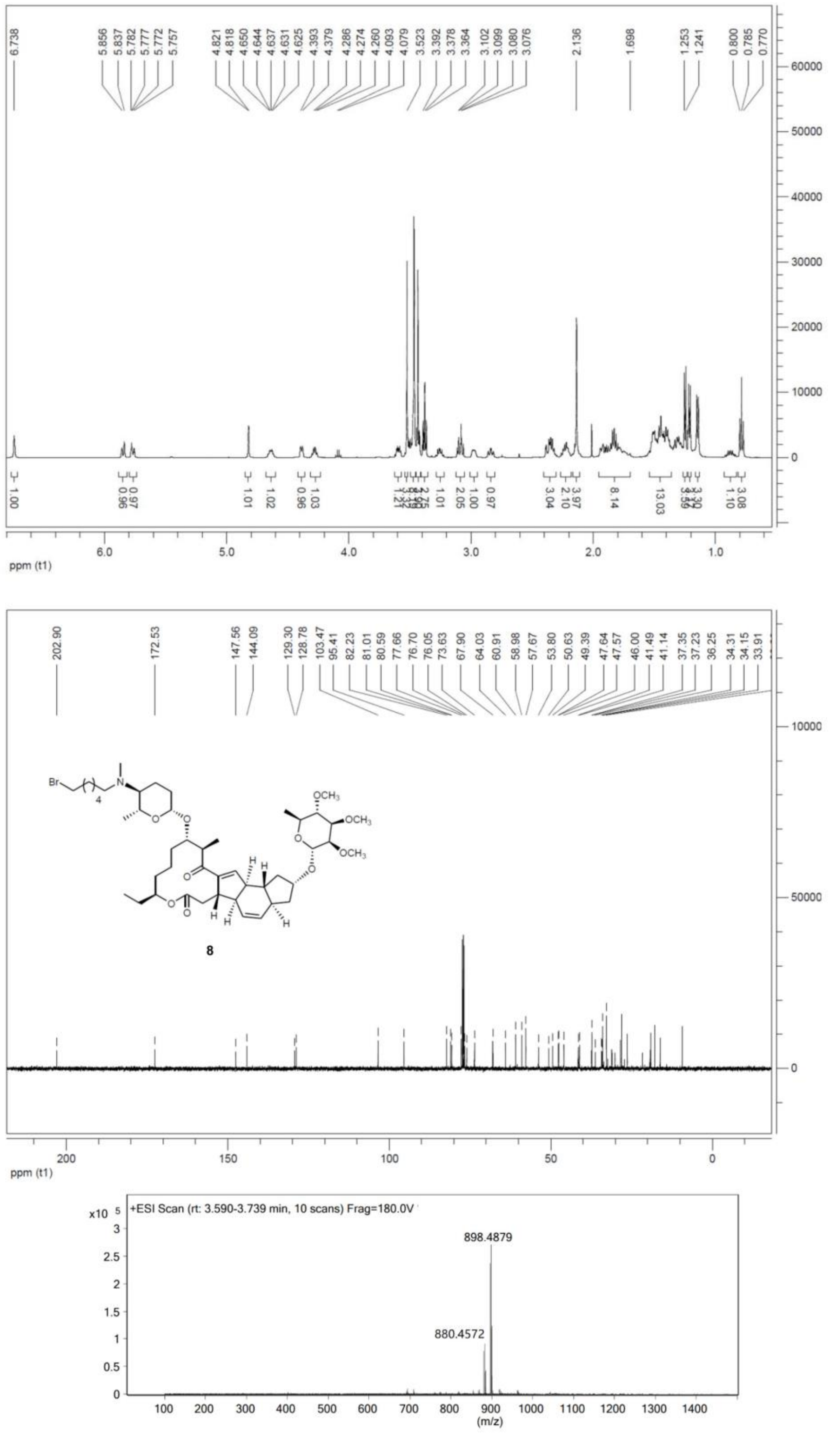

Supplementary Figure 14 | ^{1}H , ^{13}C NMR and MS spectrum for 8.

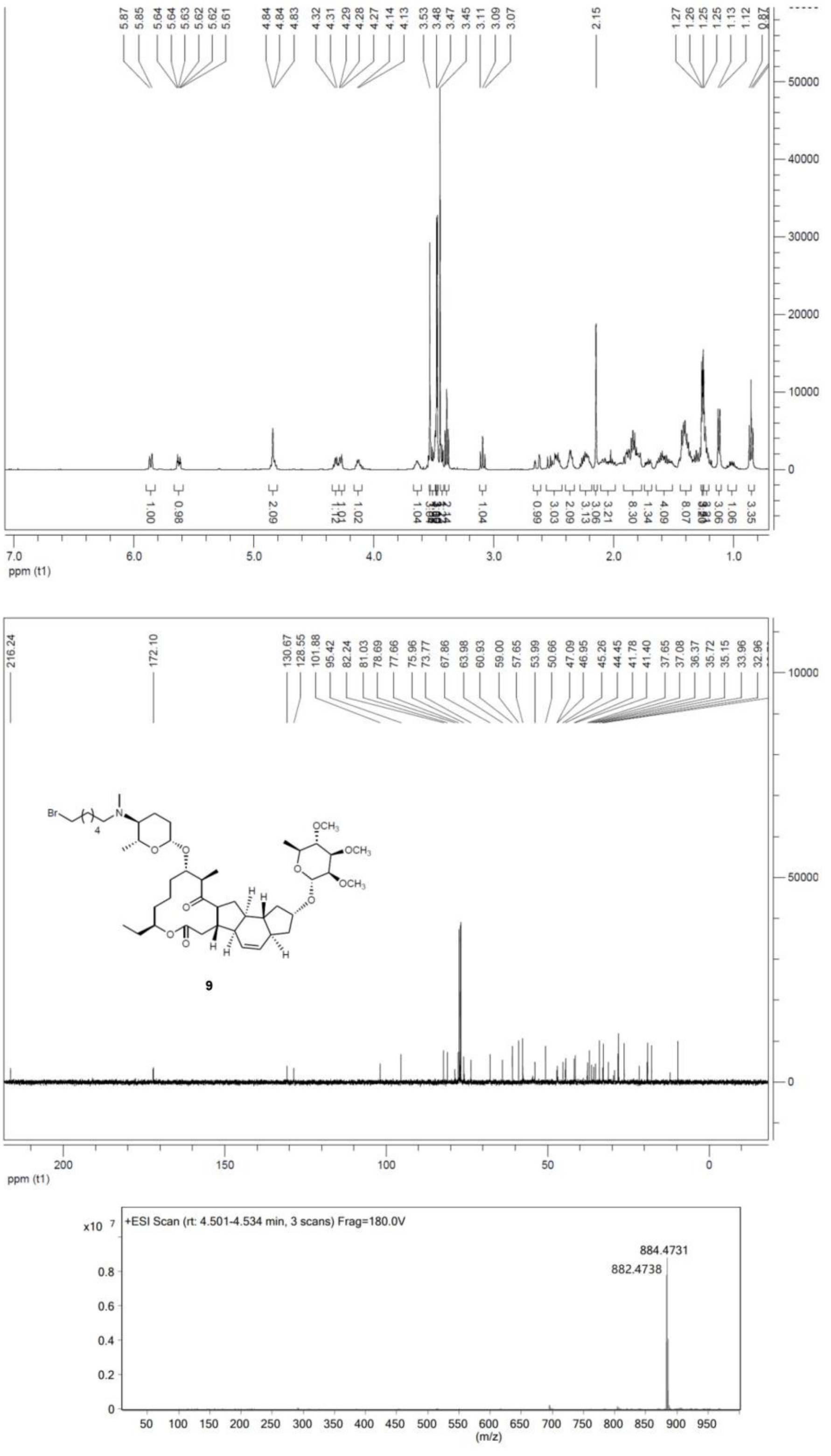

Supplementary Figure 15 | ^{1}H , ^{13}C NMR and MS spectrum for 9.
