# Supplementary material for: Naturally-occurring spinosyn A and its derivatives function as argininosuccinate synthase activator and tumor inhibitor
Source: Nat Commun. 2021 Apr 15;12:2263. doi: 10.1038/s41467-021-22235-8 (PMC8050083; doi:10.1038/s41467-021-22235-8)
Supplement: Supplementary file 3 — Reporting summary [file 41467_2021_22235_MOESM3_ESM.pdf]

nature research

Corresponding author(s): Zhiyong Luo, Junli Luo, Suyou Liu

Last updated by author(s): Feb 15, 2021

Reporting Summary

Nature Research wishes to improve the reproducibility of the work that we publish. This form provides structure for consistency and transparency in reporting. For further information on Nature Research policies, see Authors & Referees and the Editorial Policy Checklist .

Statistics

For all statistical analyses, confirm that the following items are present in the figure legend, table legend, main text, or Methods section.

n/a	Confirmed	
☐	☒	The exact sample size (*n*) for each experimental group/condition, given as a discrete number and unit of measurement
☐	☒	A statement on whether measurements were taken from distinct samples or whether the same sample was measured repeatedly
☐	☒	The statistical test(s) used AND whether they are one- or two-sided<br>*Only common tests should be described solely by name; describe more complex techniques in the Methods section.*
☒	☐	A description of all covariates tested
☒	☐	A description of any assumptions or corrections, such as tests of normality and adjustment for multiple comparisons
☐	☒	A full description of the statistical parameters including central tendency (e.g. means) or other basic estimates (e.g. regression coefficient) AND variation (e.g. standard deviation) or associated estimates of uncertainty (e.g. confidence intervals)
☐	☒	For null hypothesis testing, the test statistic (e.g. *F*, *t*, *r*) with confidence intervals, effect sizes, degrees of freedom and *P* value noted<br>*Give P values as exact values whenever suitable.*
☒	☐	For Bayesian analysis, information on the choice of priors and Markov chain Monte Carlo settings
☒	☐	For hierarchical and complex designs, identification of the appropriate level for tests and full reporting of outcomes
☒	☐	Estimates of effect sizes (e.g. Cohen's *d*, Pearson's *r*), indicating how they were calculated

Our web collection on statistics for biologists contains articles on many of the points above.

Software and code

Policy information about availability of computer code

Data collection	Leica DMshare (v3), MultiQuant software (v3.0.2, AB) , BIAevaluation (v4.1) , EnSpire software (v4.1) , Image lab (v4.1) ,Leica TCS SP8 X&MP laser scanning confocal microscope software (LASX V3.5.2.18963) .
Data analysis	Analyst (v1.6.3, AB) , SPSS 18.0 , GraphPad Prism (v8.0) , Origin (v2018, 64 Bit) , Image J (v1.48) , MOE (2018) , BIAevaluation (v4.1) .

For manuscripts utilizing custom algorithms or software that are central to the research but not yet described in published literature, software must be made available to editors/reviewers. We strongly encourage code deposition in a community repository (e.g. GitHub). See the Nature Research guidelines for submitting code & software for further information.

Data

Policy information about availability of data

All manuscripts must include a data availability statement. This statement should provide the following information, where applicable:

- Accession codes, unique identifiers, or web links for publicly available datasets
- A list of figures that have associated raw data
- A description of any restrictions on data availability

Data availability

All data supporting the findings of this study are available from the corresponding authors upon reasonable request. The atomic coordinates and structue factors of ASS1 have been deposited in the protein data bank, www.wwpdb.org (PDB code: 2NZ2) ,The uncropped gel or blot figures and original data underlying Figs. 1–6 and Supplementary Figs. 4–8 are provided as a Source Data file. The synthesis routes and analytical spectra of the chemical derivatives presented in Fig. 1-2 are provided in the Supplementary Information document. The full mass spectrometry results of pull-down assay in excel form and raw metabolomic data are presented in Source date. Source data are provided with this paper.

Field-specific reporting

Please select the one below that is the best fit for your research. If you are not sure, read the appropriate sections before making your selection.

☒ Life sciences ☐ Behavioural & social sciences ☐ Ecological, evolutionary & environmental sciences

For a reference copy of the document with all sections, see nature.com/documents/nr-reporting-summary-flat.pdf

Life sciences study design

All studies must disclose on these points even when the disclosure is negative.

Sample size	All tests were performed with n≥3 independent experiments. In vivo experiments included data from n=7-8 mice for each group and presented as mean with deviation in the graphs and figure legends. Sample sizes and statistical data are reported in figure legends.
Data exclusions	No data were excluded from analysis
Replication	Experiments were repeated with same conditions and obtained similar results. The number of repeats were indicated in figure legends.
Randomization	The mice were randomly assigned into different experimental groups whenever possible, except in experiments required specific genotypes.In different genotype groups, the average weight and tumor burden of mice are similar, and the numbers are the same
Blinding	The investigators were blinded to group allocation during data collection and analysis.

Reporting for specific materials, systems and methods

We require information from authors about some types of materials, experimental systems and methods used in many studies. Here, indicate whether each material, system or method listed is relevant to your study. If you are not sure if a list item applies to your research, read the appropriate section before selecting a response.

Materials & experimental systems

n/a	Involved in the study	
☐	☒	Antibodies
☐	☒	Eukaryotic cell lines
☒	☐	Palaeontology
☐	☒	Animals and other organisms
☐	☒	Human research participants
☒	☐	Clinical data

Methods

n/a	Involved in the study	
☒	☐	ChIP-seq
☒	☐	Flow cytometry
☒	☐	MRI-based neuroimaging

Antibodies

Antibodies used	Anti-ASS1 (Cell Signaling Technology, CAT 70720, Clone D4O4B, Lot 1, Dilution 1:1000)<br>anti-GAPDH (Cell Signaling Technology, CAT 2118, Clone 14C10, Lot 2, Dilution 1:5000)<br>Anti-Ki-67 (Cell Signaling Technology, CAT 9449, Clone 8D5, Lot 1, Dilution 1:250-500)<br>anti-rabbit IgG-HRP (Cell Signaling Technology, CAT 7074, Lot 2, Dilution 1:5000)<br>streptavidin-HRP (Cell Signaling Technology, CAT 3999, Lot 7, Dilution 1:1000)<br>Streptavidin-FITC (eBioscience, CAT 11-4317-87, Lot E00564-1634, Dilution 1:1000)<br>goat anti rabbit IgG-CY3 (Jackson ImmunoResearch, CAT 111-167-003, Dilution 1:400)
Validation	The specificity of the antibodies purchased from commercial sources (Cell Signaling Technology, eBioscience and Jackson ImmunoResearch) were validated by the manufacturer as noted on their website (links provided below for each antibody)<br>Anti-ASS1, Human, Mouse and Rat, WB, IP, IHC, IF, (https://www.cellsignal.com/products/primary-antibodies/ass1-d4o4b-xp-rabbit-mab/70720) ;<br>anti-GAPDH, Human, Mouse , Rat, Monkey, Bovine and Pig, WB, IHC, IF, F, (https://www.cellsignal.com/products/primary-antibodies/gapdh-14c10-rabbit-mab/2118) ;<br>Anti-Ki-67, Human, IHC, IF, F, (https://www.cellsignal.com/products/primary-antibodies/ki-67-8d5-mouse-mab/9449) ;<br>anti-rabbit IgG-HRP, WB, (https://www.cellsignal.com/products/secondary-antibodies/anti-rabbit-igg-hrp-linked-antibody/7074) ;<br>streptavidin-HRP, WB, (https://www.cellsignal.com/products/wb-ip-reagents/streptavidin-hrp/3999) ;<br>Streptavidin-FITC, Flow Cyt, ICC, IF, (https://www.thermofisher.com/order/catalog/product/11-4317-87?SID=srch-hj-11-4317-87#/11-4317-87?SID=srch-hj-11-4317-87) ;<br>goat anti rabbit IgG-CY3, Flow Cyt, ICC, IF, (https://www.jacksonimmuno.com/catalog/products/111-167-003) .

Eukaryotic cell lines

Policy information about cell lines

Cell line source(s)	MDA-MB-231 cells (American Type Culture Collection, HTB-26);<br>MDA-MB-468 cells (American Type Culture Collection, HTB-132);<br>Hs578T cells (American Type Culture Collection, HTB-126);<br>BT20 cells (American Type Culture Collection, HTB-19);<br>BT549 cells (American Type Culture Collection, HTB-122);<br>HCC1806 cells (American Type Culture Collection, CRL-2335);<br>HCC1937 cells (American Type Culture Collection, CRL-2336);<br>MCF-7 cells (American Type Culture Collection, HTB-22);<br>HCC1500 cells (American Type Culture Collection, CRL-2329);<br>T47D cells (American Type Culture Collection, HTB-133;<br>BT474 cells (American Type Culture Collection, HTB-20);<br>SKBR3 cells (American Type Culture Collection, HTB-30) ;<br>MCF-10A cells (American Type Culture Collection, CRL-10317) ;<br>184B5 cells (American Type Culture Collection, CRL-8799).<br>HEK293T cells (American Type Culture Collection, CRL-3216)
Authentication	Cell lines were authenticated using GenePrint STR Authentication Kit
Mycoplasma contamination	Cell lines were tested negative for mycoplasma by the supplier
Commonly misidentified lines<br>(See ICLAC register)	No commonly misidentified lines used in this study

Animals and other organisms

Policy information about studies involving animals; ARRIVE guidelines recommended for reporting animal research

Laboratory animals	4-6 week old female BALB/c nu/nu (Hunan SJA laboratory Animal CO.,LTD, Hunan, China). Animals were housed in groups of 4-6 mice per individually ventilated cage in a 12 h light/dark cycle (07:30-19:30 light; 19:30-7:30 dark), with controlled room temperature (23 ± 2°C) and relative humidity (40-50 %).
Wild animals	This study did not involve wild animals.
Field-collected samples	This study did not involve field-collected samples.
Ethics oversight	All animals were maintained in an Association for Assessment and Accreditation of Laboratory Animal Care-approved animal facility at Department of laboratory Animals of Central South University. Procedures were approved by the Institutional Animal Care and Use Committee of Central South University and were in compliance with all relevant ethical regulations.

Note that full information on the approval of the study protocol must also be provided in the manuscript.

Human research participants

Policy information about studies involving human research participants

Population characteristics	Clinicopathological characteristics of breast cancer patients are list in Supplementary Table 1.
Recruitment	Breast cancer tissues were obtained from the Second Xiangya Hospital of Central South University.
Ethics oversight	Informed consent was obtained from all participants in accordance with the Declaration of Helsinki. All protocols using human specimens were approved by the institutional Review Board of the Second Xiangya Hospital of Central South University.

Note that full information on the approval of the study protocol must also be provided in the manuscript.
